# Central States Dental Association

**Published:** 1866-05

**Authors:** 


### CENTRAL STATES DENTAL ASSOCIATION.

The semi-annual meeting of this society, was held in Nashville, Tenn., on the 10th of April. Its sessions continued three days, evening sessions were also held. There were about forty members of the profession present, all of whom seemed to enjoy the meeting very much.

We have scarcely been present at any professional gathering, where so much interest and earnestness was manifested as here. Nearly all engaged in the discussion of the topics presented. Our brethren in that section of the country, are almost wholly absorbed in professional matters, and manifest a determination to move on in the direction of progress, fully abreast with any others; and now that anarchy is removed, and order reigns in our country, our whole brotherhood will move on as a united band, pressing forward every enterprise and object that shall tend to develope and establish our noble profession.

We shall give the proceedings and discussions in the next number of the *Register*. The next meeting of this association will be held in Louisville, commencing on Tuesday, the 17th of July next, when, we doubt not, there will be a large gathering of the profession.

